# Land Use Conversion Regulates Soil Nutrient–Metal Coupling and Bacterial Diversity Through Ecological Mechanisms in Mine Reclaimed Ecosystems

**DOI:** 10.3390/plants15182824

**Published:** 2026-09-15

**Authors:** Hui Hu, Yupeng Sang, Tingting Zhang, Yu Yang, Zhenyuan Huang, Kaibin Qi

**Affiliations:** 1 Henan Key Laboratory of Water Pollution Control and Rehabilitation, Henan University of Urban Construction, Pingdingshan 467041, China; 20221063@huuc.edu.cn (H.H.);; 2 Institute of Mountain Hazards and Environment, Chinese Academy of Sciences, Chengdu 610213, China; yangyu@imde.ac.cn; 3 Academy of Agriculture and Forestry Sciences, Qinghai University, Xining 810016, China

**Keywords:** land use change, soil nutrient cycling, heavy metal accumulation, microorganism diversity, piecewise structural equation modeling

## Abstract

Land use conversion profoundly affects the ecological functioning of reclaimed soils. However, the coupling mechanisms among soil nutrient accumulation, heavy metal dynamics, and microbial diversity under different land use types remain unclear. This study investigated reclaimed mining soils under five land use types to evaluate changes in soil properties, heavy metal distribution, and bacterial diversity, and to elucidate their regulatory mechanisms. Wheat fields and Ligustrum plantations showed higher carbon, nitrogen, and phosphorus contents, with increased lead and arsenic concentrations, whereas abandoned grasslands exhibited relatively lower nutrient availability. Land use explained 26.4% of bacterial community variation (R^2^ = 0.264, *p* = 0.001). Structural equation modeling revealed that nutrient enrichment significantly promoted heavy metal accumulation (β = 0.606, *p* < 0.001). Nutrients negatively affected bacterial richness (β = −0.401, *p* = 0.035). In contrast, moderate metal stress may promote microbial richness (β = 0.460, *p* = 0.015) through micronutrient effects or hormesis (low-dose toxic stimulatory response). These findings indicate that land use conversion regulates microbial diversity through coupled changes in nutrient availability and metal dynamics. Reclamation strategies should integrate nutrient management, vegetation selection, and heavy metal risk assessment to achieve long-term restoration of soil functions.

## 1. Introduction

Coal mining has caused extensive land degradation, vegetation loss, and heavy metal contamination, making mine reclamation essential for restoring soil functions and ecosystem services [1,2,3,4]. The early reclamation stage (5–10 years) is a critical period when soil–vegetation interactions and emerging geochemical gradients shape subsequent ecosystem development [5,6]. Different land use types can establish contrasting ecological states because of differences in vegetation composition, resource inputs, and management intensity [6]. Vegetation type regulates soil structure and nutrient dynamics through variation in litter stoichiometry, root architecture, exudates, tillage, irrigation, and fertilization [6,7,8]. Abandoned grasslands can increase soil organic carbon (SOC) and aggregate stability, although recovery may be slow because of limited species availability [9]. Intensive agricultural use may increase soil compaction and reduce organic matter accumulation through frequent tillage and crop harvesting [10,11], whereas forest vegetation can promote carbon sequestration and improve soil structure through deep rooting and litter inputs [11]. Deciduous trees may also facilitate early soil recovery through rapid growth and efficient nutrient acquisition [12,13]. Intensive vegetable cultivation may accelerate soil acidification and nutrient imbalance because of frequent irrigation and high fertilizer inputs [14]. These land use-driven gradients in physical properties and inorganic nutrients can shape soil fertility trajectories and further regulate heavy metal retention, speciation, and mobility through organic matter–mineral interactions.

Soil physical and chemical properties are fundamental factors determining the success of ecological restoration in mining areas. Soil physical parameters, including bulk density, porosity, and water content, directly regulate water transport, gas exchange, and root penetration resistance [15,16]. Excessive bulk density and poorly developed aeration pore networks can severely restrict microbial metabolic activity and nutrient turnover processes [17]. From a chemical perspective, soil pH, organic carbon, and nitrogen and phosphorus availability are key indicators of soil fertility recovery and serve as critical geochemical regulators controlling heavy metal adsorption–desorption and precipitation–dissolution processes [18]. SOC functions as a major carbon reservoir and, meanwhile, as an essential energy source supporting microbial activity and nutrient cycling [19]. Soil ecological stoichiometry provides insights into nutrient balance and potential nutrient limitations in terrestrial ecosystems [20,21]. Specifically, the C:N ratio reflects organic matter decomposition and nitrogen supply conditions. The N:P ratio is commonly used to assess potential nutrient limitations in terrestrial ecosystems. In addition to nutrient recovery, residual heavy metals remain a major constraint on ecological restoration in mining areas. Mining activities can promote the long-term accumulation of elements such as cadmium (Cd), lead (Pb), arsenic (As), and mercury (Hg) in soils, which may impair plant growth, disrupt soil biological processes, and reduce ecosystem stability [22]. However, heavy metal ecological risks are not solely determined by their total concentrations. Their mobility and bioavailability are jointly regulated by soil pH, organic matter content, and other environmental conditions [23]. This creates a complex interplay in which physical constraints and nutrient dynamics jointly influence heavy metal accumulation and retention. Therefore, simultaneous assessment of nutrient recovery and heavy metal dynamics is essential for accurately evaluating reclamation effectiveness. Nevertheless, previous studies have mainly focused on individual soil properties or specific heavy metal elements, while the cascading effects and synergistic mechanisms among physical constraints, nutrient accumulation, and heavy metal retention remain poorly understood. Given the high heterogeneity of soil properties during the early reclamation stage, integrating multiple environmental variables and their interactions is urgently required to accurately evaluate reclamation outcomes.

Soil bacterial communities serve as key biological engines driving nutrient cycling, organic matter decomposition, and heavy metal transformation and detoxification. Their diversity is widely recognized as a sensitive bioindicator of soil health and ecosystem resilience under environmental stress [24,25]. In mining areas undergoing reclamation, vegetation restoration can reshape bacterial community structure by altering litter quality, root exudates, and microhabitat conditions [26,27]. Previous studies have consistently demonstrated that soil nutrient availability is one of the most reliable predictors of bacterial diversity patterns. Nutrient conditions regulate microbial community assembly by controlling metabolic substrate supply and ecological niche differentiation [28]. Meanwhile, heavy metal stress can inhibit the establishment and proliferation of sensitive bacterial taxa, leading to reduced microbial diversity and simplified ecological networks [29]. In mining ecosystems, improvements in soil physical structure and nutrient accumulation provide favorable habitats for microbial colonization, whereas residual heavy metals impose persistent environmental filtering pressure. These opposing effects may generate complex antagonistic and synergistic interactions that jointly determine microbial recovery trajectories [30,31]. Therefore, the recovery of bacterial diversity not only reflects the direct ecological effectiveness of reclamation practices but also provides a biological perspective for evaluating the actual restoration status of soil ecosystems. However, most existing studies have focused on single-factor relationships, and the integrated cascading pathways linking vegetation, soil physicochemical properties, heavy metals, and microbial communities remain insufficiently understood.

**Figure 1:**
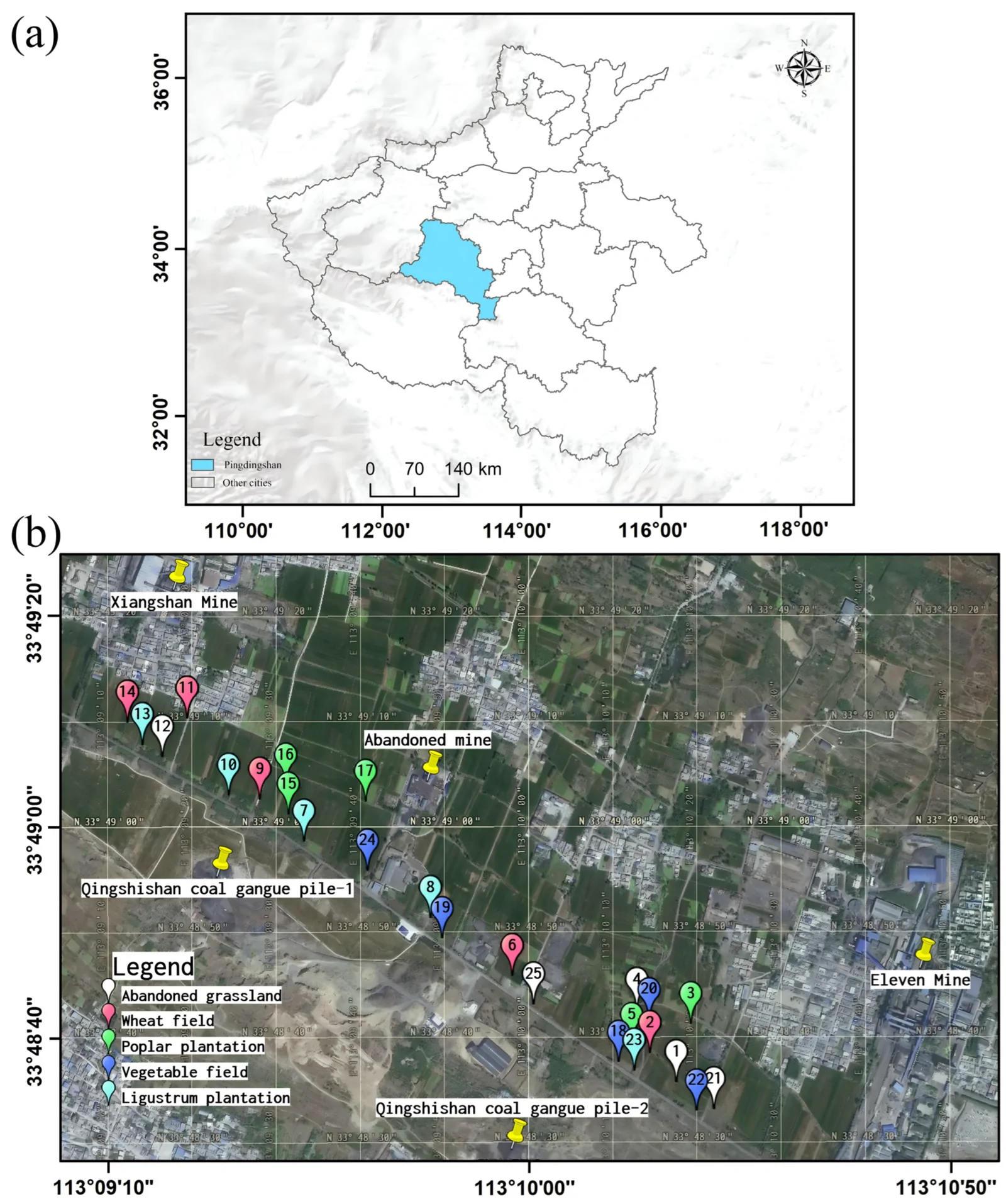
Location of the study area and sampling sites. (**a**) Location of Pingdingshan City in Henan Province, China; (**b**) spatial distribution of the 25 sampling sites across the five land use types in the reclaimed mining area.

Although numerous studies have separately investigated the effects of vegetation restoration on soil properties or heavy metal dynamics in mining reclamation [7,32,33,34], a comprehensive causal framework that integrates land use types as external drivers and links soil physical structure, nutrient stoichiometry, multi-element heavy metal profiles, and bacterial diversity remains largely unexplored. In this study, five representative land use types established six years after mine reclamation were selected (Figure 1), including naturally restored abandoned grassland, wheat–maize rotation cropland, poplar plantation, Ligustrum plantation, and vegetable rotation cropland. We systematically characterized soil physical properties, nutrient contents, total concentrations of nine heavy metals, and bacterial diversity based on 16S rRNA gene sequencing. The objectives of this study were to: (i) quantify how different land use types alter soil physical properties, nutrient availability, and heavy metal accumulation across soil depths; (ii) identify the major patterns of nutrient and heavy metal differentiation among land use types and determine their associations with bacterial diversity; and (iii) disentangle the direct and indirect pathways through which land use conversion regulates soil heavy metal accumulation and bacterial diversity using a piecewise structural equation model (SEM). The findings provide mechanistic insights into optimizing vegetation configuration strategies and improving long-term ecological management in reclaimed mining ecosystems.

## 2. Results

### 2.1. Soil Basic Properties

Different land use types significantly altered soil physical properties and moisture content (Table A2). In the 0–10 cm soil layer, soil moisture content under the Wheat field (WF) treatment was significantly higher than that under the Poplar plantation (PP), Vegetable field (VF), and Abandoned grassland (AG) (*p* < 0.05), whereas the Ligustrum plantation (LP) showed significantly higher moisture content only compared with AG (Table 1). Soil bulk density was highest in AG and was significantly greater than that in VF and LP (Table 1). Total porosity reached the highest value in VF, with a significant difference observed only between VF and AG (*p* < 0.05).

In the 10–20 cm soil layer, the variation patterns of soil moisture content, bulk density, and total porosity were generally consistent with those observed in the surface soil layer, although the magnitude of differences among treatments was reduced (Table 1). In contrast, capillary porosity and non-capillary porosity at this depth showed significant differences among land use treatments. Across all five land use types, soil moisture content and bulk density in the 0–10 cm layer were generally lower than those in the 10–20 cm layer, whereas soil porosity and pH exhibited the opposite pattern (Table 1). Soil water content, total porosity, and capillary porosity significantly differed among soil layers. (Table A2).

### 2.2. Soil Nutrients and Stoichiometric Characteristics

**Figure 2:**
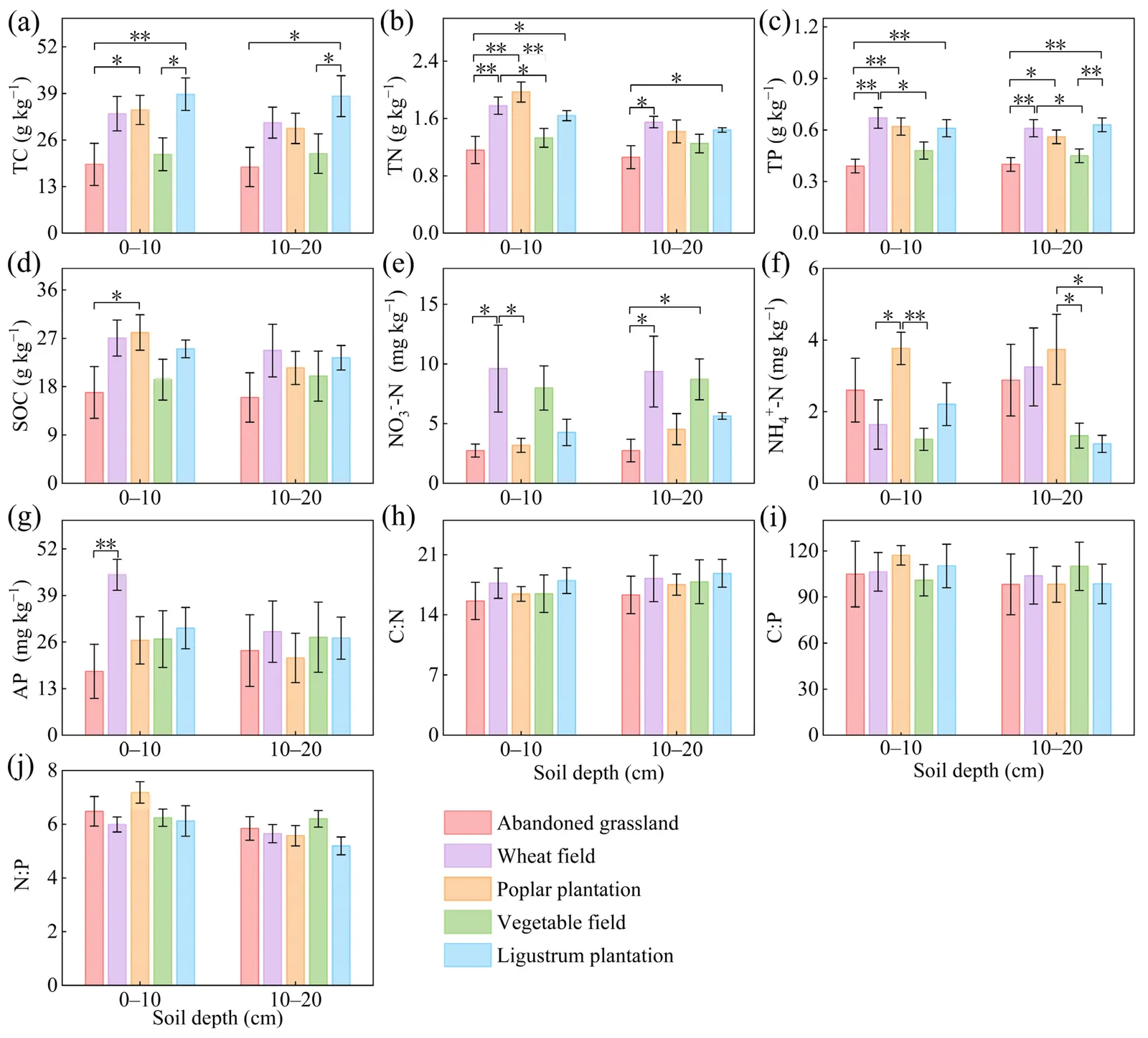
Differences in soil nutrient content and stoichiometric ratio among five land use types. Asterisks indicate significant differences among land use types: *p* < 0.05 (*) and *p* < 0.01 (**). Differences in soil total carbon (**a**), total nitrogen (**b**), total phosphorus (**c**), organic carbon (**d**), nitrate nitrogen (**e**), ammonium nitrogen (**f**), available phosphorus (**g**), C:N (**h**), C:P (**i**) and N:P (**j**) among land use types across two soil layers.

Different land use types significantly altered soil nutrient contents (Table A1). In the 0–10 cm soil layer, Soil total carbon (TC) content was highest in the LP and was significantly higher than that in VF and AG (*p* < 0.05, Figure 2a). Soil total nitrogen (TN) and soil organic carbon (SOC) content exhibited similar patterns, with higher concentrations observed in WF and PP, whereas lower concentrations were found in VF and AG treatments (Figure 2b,d). Total phosphorus (TP) content was highest in WF and was significantly greater than that in VF and AG (*p* < 0.05). NO_3^−^_-N showed the highest concentration in WF, which was significantly higher than that in AG and PP (Figure 2e). NH_4^+^_-N content was highest in PP and was significantly greater than that in WF and VF (*p* < 0.05). Available phosphorus (AP) content also reached the highest level in WF and was significantly higher than that in AG (Figure 2g). The nutrient stoichiometric ratios showed relatively small variations among the five land use types. Although N:P and C:P ratios in PP were slightly higher than those in the other treatments with not statistically significant (Figure 2h–j). In the 10–20 cm soil layer, the concentrations of most nutrient contents showed an overall decreasing trend compared with the surface layer, and the differences among land use treatments were reduced (Figure 2). Soil TN and the N:P ratio exhibited significant variations (*p* < 0.05) across soil depths (Table A2).

**Figure 3:**
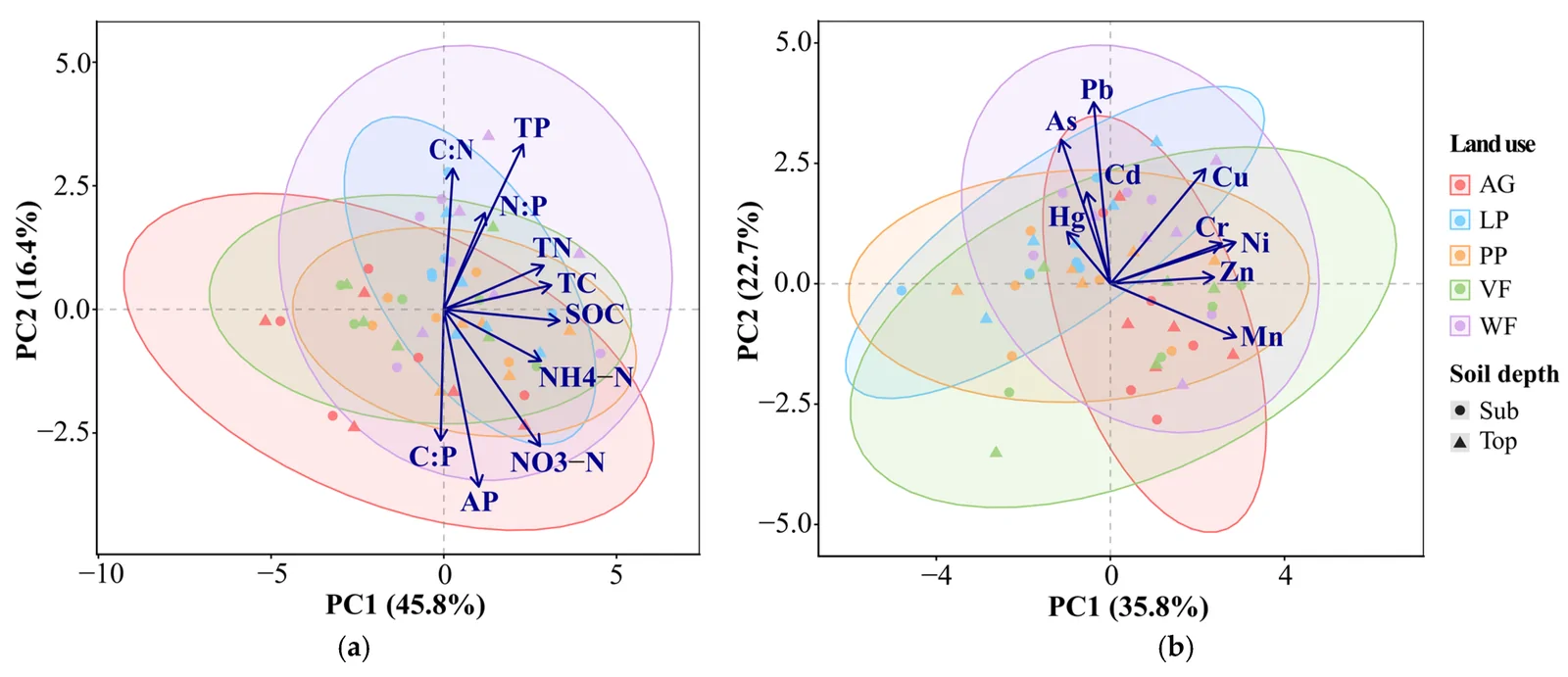
Principal component analysis (PCA) of soil nutrient status (**a**) and heavy metal concentrations (**b**) among different land-use types in the 0–10 cm and 10–20 cm soil depth. AG: Abandoned grassland, LP: Ligustrum plantation, PP: Poplar plantation, WF: Wheat field, VF: Vegetable field.

PCA further revealed distinct nutrient accumulation patterns among different land use types (Figure 3a). PC1 explained 45.8% of the total variance and was mainly associated with SOC, TC, TN, and NH_4^+^_-N, representing a gradient of soil carbon and nutrient accumulation. AG was separated toward the negative direction of PC1, indicating depleted carbon and nutrient conditions. In contrast, WF and LP exhibited positive PC1 scores, reflecting enhanced carbon and nitrogen accumulation. PC2 explained 16.4% of the total variance and was primarily associated with soil C:N, N:P, and C:P ratios, representing differences in nutrient balance and stoichiometric constraints. WF showed higher PC2 scores, characterized by relatively higher C:N and N:P ratios, whereas AG exhibited lower PC2 scores, which were associated with increased availability of inorganic nutrients.

### 2.3. Soil Heavy Metal Concentrations Distribution

**Figure 4:**
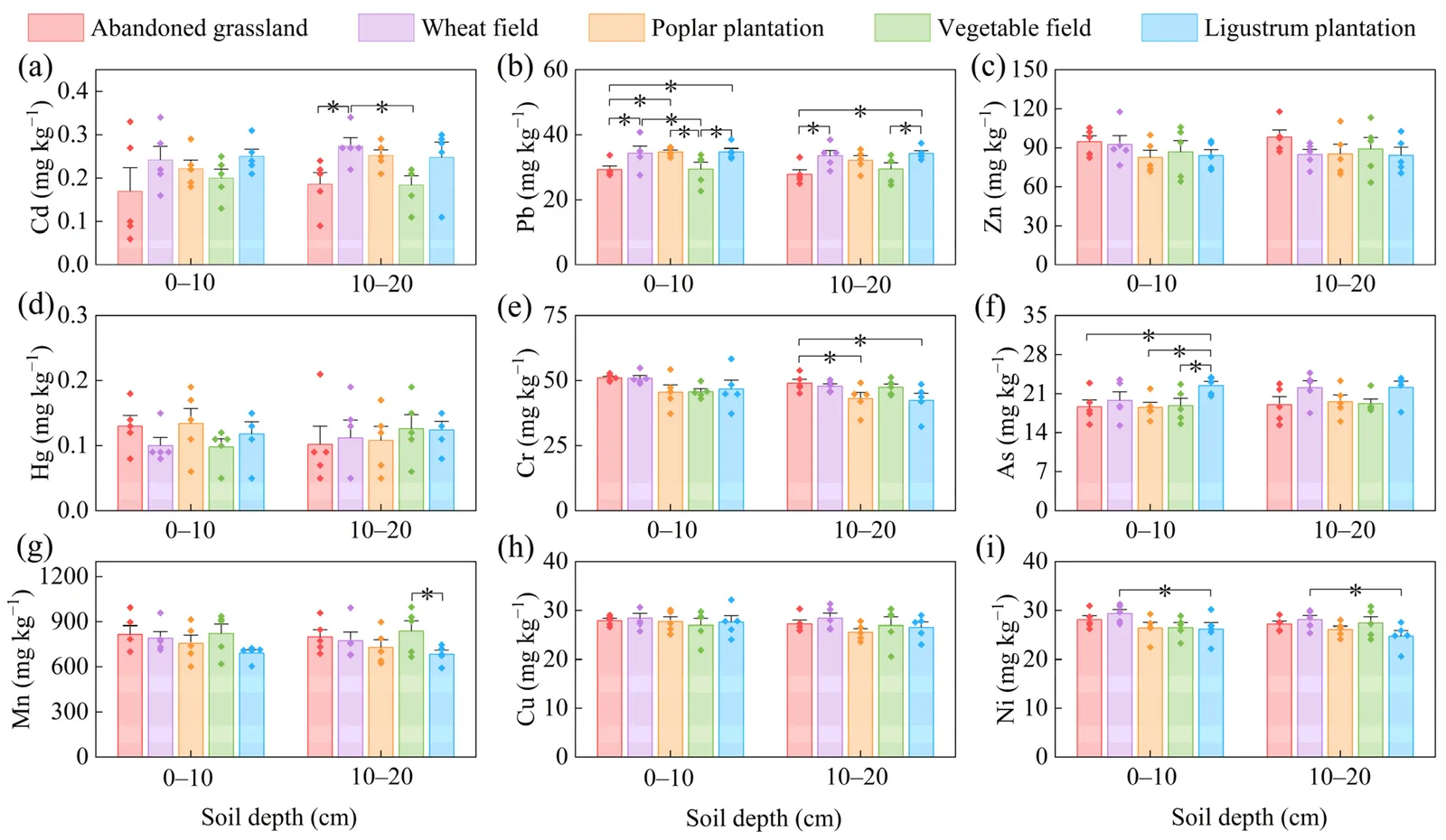
Differences in soil heavy metal content among five land use types. Asterisks indicate significant differences among land use types: *p* < 0.05 (*). Differences in soil cadmium (**a**), lead (**b**), zinc (**c**), mercury (**d**), chromium (**e**), arsenic (**f**), manganese (**g**), copper (**h**) and nickel (**i**) among land use types across two soil layers.

Different land use types significantly altered soil heavy metal concentrations (Table A2). In the 0–10 cm soil layer, cadmium (Cd) concentration was highest in the LP treatment (Figure 4a). lead (Pb) concentrations were relatively higher in the WF, PP, and LP treatments, and were significantly greater than those in AG and VF treatments (*p* < 0.05). Arsenic (As) concentration reached the highest level in LP and was significantly higher than that in VF, PP, and AG (*p* < 0.05) (Figure 4f). Nickel (Ni) concentration was highest in WF and was significantly higher than that in LP (*p* < 0.05) (Figure 4i). In the 10–20 cm soil layer, the overall variation patterns of heavy metal concentrations were generally consistent with those observed in the surface layer, although the magnitude of differences among treatments varied slightly (Figure 4). Chromium (Cr) concentration was significantly higher in AG than in PP and LP (*p* < 0.05). Manganese (Mn concentration was highest in VF and was significantly higher than that in LP (*p* < 0.05). Soil heavy metal concentrations were not significantly affected by soil depth or the interaction between soil depth and land use types (Table A2).

PCA revealed distinct heavy metal accumulation patterns among different land use types (Figure 3b). The first principal component (PC1, 35.8%) was mainly driven by zinc (Zn), Ni, and Cr, representing a Zn–Ni–Cr enrichment gradient. AG was positioned toward the positive direction of the PC1 axis, indicating enhanced accumulation of these metals, whereas PP and LP exhibited relatively lower levels of Zn- and Ni-associated metals. The second principal component (PC2, 22.7%) was primarily associated with Pb, As, and Cd, representing a Pb–As contamination gradient. WF and some LP soils showed stronger associations with Pb and As, suggesting potential accumulation of these elements. Overall, land use conversion significantly altered soil heavy metal assemblages, resulting in distinct patterns of metal accumulation across different ecosystems.

### 2.4. Soil Microbial Diversity

**Figure 5:**
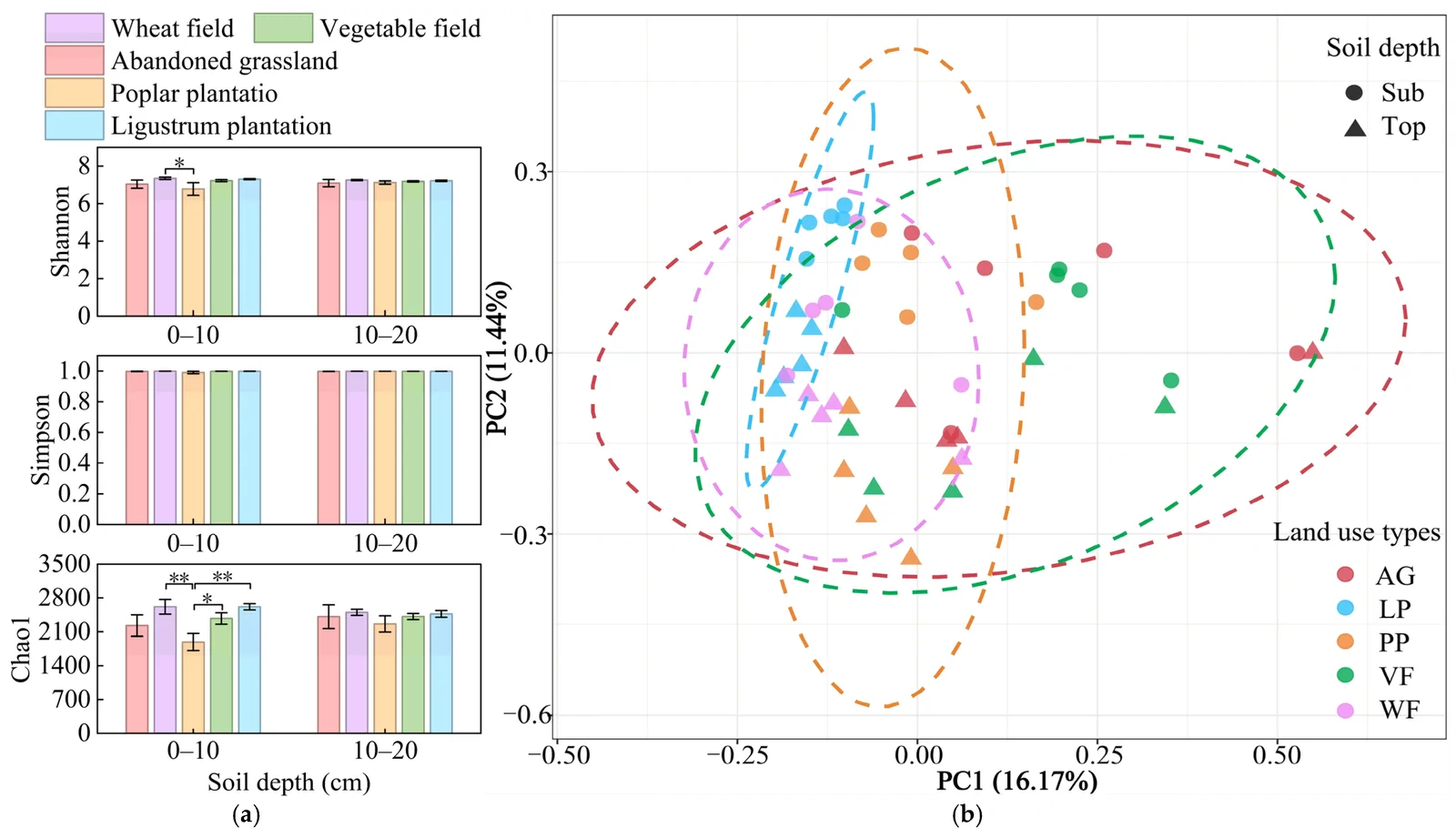
Soil bacterial α-diversity (**a**) and principal coordinate analysis (PCoA) of community composition based on Bray–Curtis distance (**b**) among different land use types in the 0–10 cm and 10–20 cm soil layers. Dashed circles represent the 95% confidence ellipses for each land use type. Land use type significantly altered microbial community composition, explaining 26.4% of the total variation in Bray–Curtis dissimilarity (PERMANOVA, R^2^ = 0.264, F = 3.155, *p* = 0.001). Asterisks indicate significant differences among land use types: *p* < 0.05 (*) and *p* < 0.01 (**). AG: Abandoned grassland, LP: Ligustrum plantation, PP: Poplar plantation, WF: Wheat field, VF: Vegetable field.

Different land use types significantly affected soil bacterial α diversity (Table A2). In the 0–10 cm soil layer, the Shannon index was highest in the WF and was significantly higher than that in the PP, whereas no significant differences were observed among the other treatments (Figure 5a). The Chao1 index was lowest in PP and was significantly lower than that in the VF, WF, and LP treatments (*p* < 0.05) (Figure 5a). In the 10–20 cm soil layer, bacterial alpha diversity showed similar variation patterns to those observed in the surface soil layer, and no significant differences were detected among different land use treatments (Figure 5a). Two-way analysis of variance (Table A2) indicated that land use type significantly affected the Chao1 index (*p* < 0.05), whereas soil depth and their interaction had no significant effects on bacterial α diversity. Soil bacterial α diversity was not significantly affected by soil depth or the interaction between soil depth and land use types (Table A2).

PCoA based on Bray–Curtis distance matrices revealed distinct differences in soil bacterial community composition among different land use types (Figure 5b). The first and second PCoA axes explained 16.17% and 11.44% of the total community variation, respectively, accounting for 27.61% of the overall variation. Samples from different land-use types exhibited clear separation patterns in the two-dimensional ordination space. Specifically, samples from the VF and AG were mainly distributed along the positive direction of PCoA1, whereas samples from the LP and WF were concentrated in the negative direction of PCoA1. These patterns indicate that different land use systems developed distinct bacterial community structures. Samples from WF and PP showed considerable overlap in their distribution ranges, suggesting a relatively similar bacterial community composition between these two land use types. PERMANOVA further confirmed that land use type significantly influenced bacterial community structure (R^2^ = 0.264, F = 3.155, *p* = 0.001), indicating that land-use conversion was an important driver of soil bacterial community variation.

### 2.5. Regulatory Mechanisms of Soil Microbial Diversity

**Figure 6:**
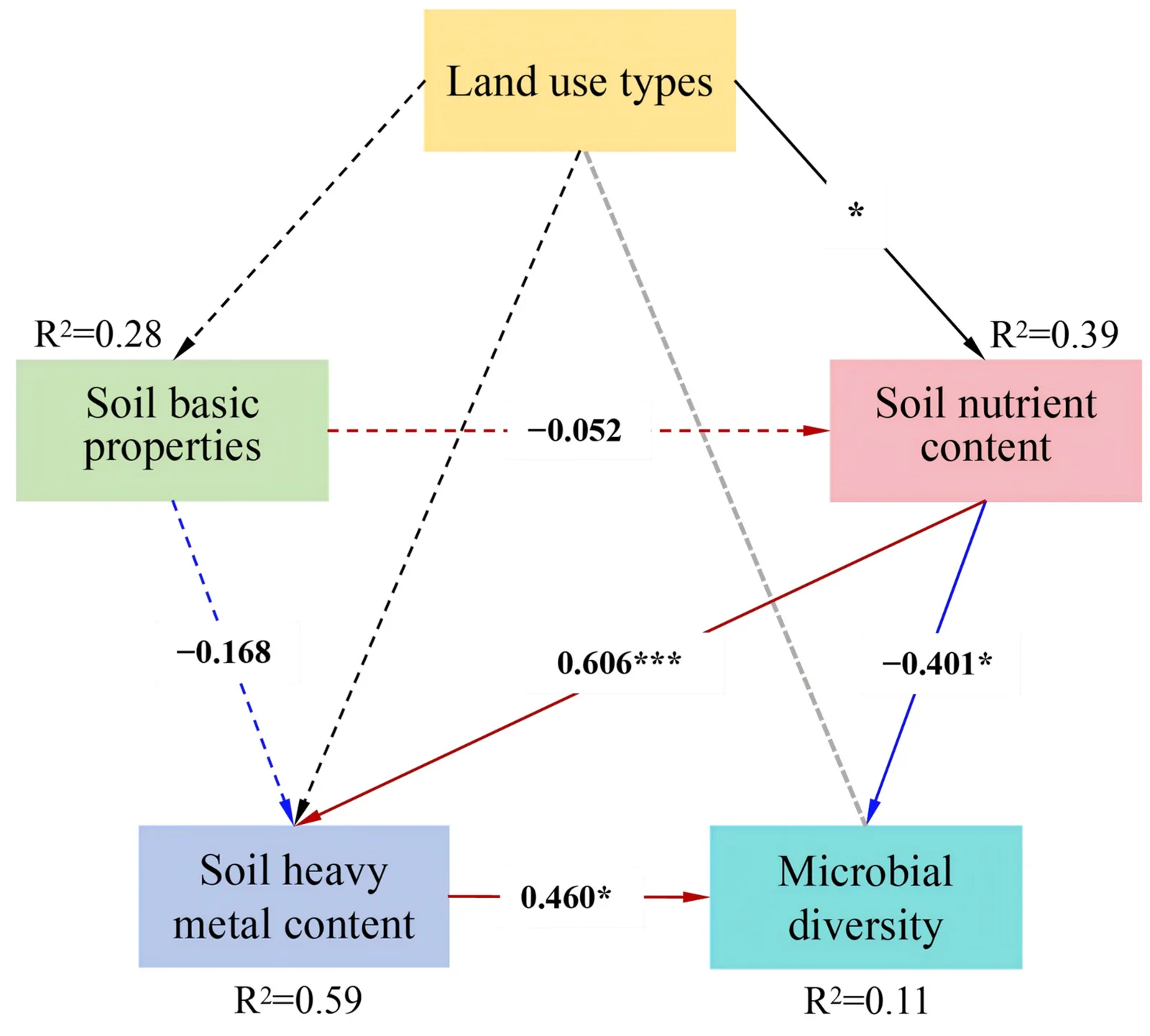
Piecewise structural equation model (piecewise SEM) illustrating the cascading effects of land-use types, basic soil properties, nutrient status, and heavy metal content on bacterial diversity. Red indicates a positive effect, and blue indicates a negative effect. * *p* < 0.05 and *** *p* < 0.001. The overall fit of the model was good (fisher’s C = 4.406, DF = 6, *p* = 0.622; AIC = 497.309). In this model, land use types are used as classification variables. Principal component analysis (PCA) was conducted for soil basic properties (bulk density, pH, water content), nutrient content (soil organic carbon, total nitrogen, total phosphorus), and heavy metal contents (Cd, Pb, As, Hg), and the first principal component (PC1) was extracted as the comprehensive index of the corresponding potential variable. Chao1 represents soil microbial diversity. The detailed path coefficient and random effect variance components are shown in Table A3, Table A4, Table A5 and Table A6, respectively.

Piecewise SEM indicated that land use types indirectly regulated bacterial richness through soil nutrient accumulation and metal enrichment (Figure 6 and Table A3, Table A4, Table A5 and Table A6). Nutrient availability was the primary driver of soil metal accumulation (β = 0.606, *p* < 0.001). However, bacterial richness showed contrasting responses to soil nutrients and metals, with nutrients negatively affecting Chao1 richness (β = −0.401, *p* = 0.035), whereas metal accumulation exhibited a positive association (β = 0.460, *p* = 0.015). The final model showed adequate goodness-of-fit (Fisher’s C = 4.406, *p* = 0.622), suggesting that the proposed causal structure sufficiently explained observed ecological relationships. Fixed effects explained 11%, 39%, 58%, and 28% of the variance in bacterial richness, nutrient status, metal accumulation, and soil physical properties, respectively (marginal R^2^). After accounting for site-level random effects, the explained variance increased to 62%, 88%, 72%, and 82%, respectively (conditional R^2^, Table A4).

**Figure 7:**
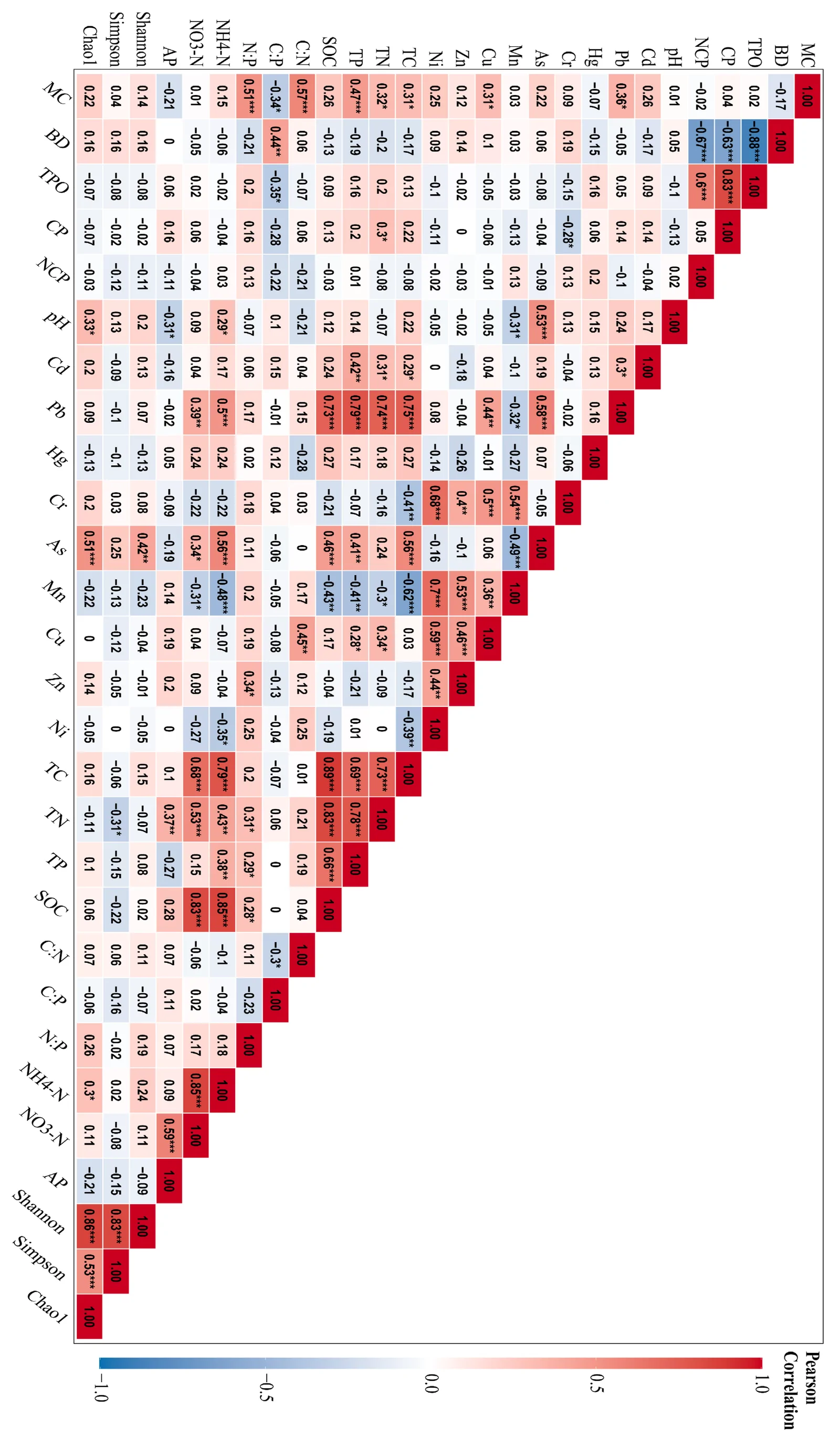
Pearson correlation heatmap among soil physicochemical properties, heavy metal concentrations, and bacterial α-diversity indices. Significance levels are denoted by asterisks: * *p* < 0.05, ** *p* < 0.01, *** *p* < 0.001. BD: bulk density, MC: moisture content, TPO: total porosity, CP: capillary porosity, NCP: non-capillary porosity, SOC: soil organic carbon, TC: total carbon, TN: total nitrogen, TP: total phosphorus, NH4-N: ammonium nitrogen, NO3-N: nitrate nitrogen, AP: available phosphorus.

The correlation heatmap also revealed the relationships among soil physicochemical properties, heavy metal concentrations, and microbial diversity (Figure 7). Soil nutrient variables, including TC, TN, TP, and SOC, showed significant positive correlations with heavy metal concentrations (Cd, Pb, and As). Bacterial diversity was significantly correlated with As concentration and TN content, indicating that nutrient availability and specific heavy metal factors may jointly regulate soil microbial diversity under different land use systems.

## 3. Discussion

### 3.1. Effects of Land Use Types on Soil Physicochemical Properties and Heavy Metal Concentrations

Different land use types substantially altered soil physical properties, nutrient availability, and stoichiometric characteristics after six years of reclamation. The Wheat field and Ligustrum plantation had the highest soil moisture contents, whereas the Vegetable field showed the highest total porosity (Table 1). In contrast, the Abandoned grassland had the highest bulk density and the lowest moisture content and total porosity, likely reflecting limited root activity and mechanical disturbance during early vegetation recovery [35]. Soil pH remained relatively stable among land use types, suggesting that it was still primarily controlled by the original parent materials and backfilled substrates, with short-term vegetation restoration having limited effects on soil acidity–alkalinity [6,28]. Soil nutrient patterns showed clearer differences among land use types. The Ligustrum and Poplar plantations exhibited higher TC, SOC, and TN contents and were positioned on the positive side of PC1, indicating greater soil carbon and nutrient accumulation capacity. This pattern may be related to continuous litter inputs, root turnover, rhizosphere carbon release, and relatively low soil disturbance under perennial woody vegetation, which favor carbon retention and nutrient cycling [21,36,37]. Continuous canopy cover may further reduce soil temperature fluctuations and evaporation, promoting microbial residue accumulation and mineral-associated organic carbon formation [38]. The Wheat field had higher TP, NO_3^−^_-N, and AP, probably owing to long-term fertilizer inputs, although the elevated C:N and N:P ratios suggested altered nutrient stoichiometry and potential nitrogen limitation [39]. In contrast, the Vegetable field showed relatively low SOC, TN, and TC despite intensive management, likely because frequent tillage, short crop cycles, and substantial nutrient removal accelerated organic matter mineralization and nutrient depletion [40]. The Abandoned grassland had the lowest SOC, TN, and TP and was located on the negative side of PC1, reflecting limited nutrient accumulation during early succession. Although natural restoration is generally recognized as an effective strategy for enhancing soil organic carbon sequestration, soil nutrient accumulation during the early restoration stage is often slow because of insufficient vegetation biomass, limited litter inputs, and underdeveloped microbial processes [41]. Overall, woody plantations favored long-term carbon and nutrient retention, agricultural management enhanced short-term nutrient availability, whereas natural restoration required a longer succession period to rebuild soil nutrient pools.

In addition to nutrient dynamics, land-use conversion significantly influenced the spatial distribution of soil heavy metals. This study found that Pb, As, Cd, and Ni exhibited pronounced differences among land-use types, whereas Zn, Cu, Hg, and Mn were less affected by land-use changes. These results indicate that different elements have distinct sources and environmental response mechanisms. The accumulation of heavy metals in soils is generally controlled by both external inputs and soil immobilization processes. Agricultural inputs, atmospheric deposition, and organic matter interactions are considered major factors regulating heavy metal retention in soils [18,42,43]. In this study, soils under the Wheat field and Ligustrum plantation exhibited higher Cd, Pb, and As concentrations, which may be associated with enhanced metal immobilization resulting from long-term nutrient inputs and organic matter accumulation. The SEM results further demonstrated that soil nutrient status had a significant positive effect on heavy metal accumulation (β = 0.606, *p* < 0.001), suggesting that nutrient enrichment processes may facilitate the retention of heavy metals in reclaimed soils. This finding supports previous studies suggesting that soils with higher fertility may possess stronger capacities for heavy metal stabilization [44]. The accumulation of soil organic matter and nitrogen and phosphorus inputs can increase the abundance of active functional groups, such as carboxyl and hydroxyl groups, providing additional adsorption sites for Cd, Pb, and As. These processes may also enhance metal–organic matter complexation, thereby reducing metal mobility and promoting total metal accumulation in soils [18,23,42]. Furthermore, long-term fertilization can alter soil redox conditions and influence heavy metal transformation among different chemical forms. For example, phosphorus fertilizer inputs may promote the formation of insoluble lead phosphate minerals, whereas increased organic matter content may enhance the immobilization of Cd and Cu through complexation processes. These findings indicate that improving soil fertility during agricultural management or vegetation restoration does not necessarily correspond to simultaneous improvements in ecological quality. Long-term fertilization, plant residue inputs, and organic matter accumulation may enhance soil colloidal adsorption capacity and increase the potential for heavy metal immobilization [18]. Meanwhile, external inputs of nutrients and contaminants may jointly modify soil elemental cycling processes, potentially resulting in a “high fertility–high heavy metal accumulation” ecological risk pattern [18,44].

Heavy metal sources varied among different land use systems. The higher Pb and Ni concentrations observed in the Wheat field may be associated with long-term agricultural inputs, including phosphate fertilizers, pesticides, and atmospheric deposition. Previous studies have shown that phosphate fertilizers may contain trace impurities such as Pb and Cd, and their long-term application can result in the gradual accumulation of these elements in agricultural soils [42,45]. Meanwhile, the elevated As concentration in the Ligustrum plantation may be related to vegetation-mediated metal retention and increased soil organic matter accumulation. The root systems of woody plants can modify rhizosphere redox conditions and promote the immobilization of As through interactions with organic ligands on soil surfaces [46]. However, long-term vegetation cover may also reduce soil disturbance, allowing historically introduced heavy metals to gradually accumulate in surface soil layers. The measured heavy metal concentrations were below the risk screening values specified in the Chinese Soil Environmental Quality–Risk Control Standard for Soil Contamination of Agricultural Land (Trial) (GB 15618–2018, Table A7, pH > 7.5) [47], indicating that soils in the study area remained within an overall safe range. However, As concentrations in some samples from the Ligustrum plantation approached the screening threshold of 25 mg kg^−1^. This finding suggests that the potential risk of As accumulation under long-term woody vegetation cover and continuous nutrient inputs should not be overlooked and should be incorporated into future monitoring programs. Furthermore, PCA revealed distinct patterns of heavy metal assemblages among different land use types (Figure 3). PC1 mainly represented a Zn–Ni–Cr accumulation gradient. The Abandoned grassland was positioned toward the positive direction of PC1, indicating stronger associations with Zn- and Ni-related accumulation characteristics. In contrast, the Poplar plantation and Ligustrum plantation were located in areas with relatively lower levels of these metals, which may be related to the enhanced metal uptake and immobilization capacity of natural recovery processes and woody vegetation. In comparison, PC2 was primarily controlled by Pb, As, and Cd. The Wheat field and Ligustrum plantation showed stronger associations with this axis, further supporting the important roles of agricultural inputs and long-term vegetation establishment in Pb and As accumulation. Overall, this study demonstrates that land use practices regulate soil carbon inputs, nutrient cycling, and chemical environments, thereby generating distinct nutrient–metal coupling patterns. These coupled processes may further influence soil microbial community diversity and ecosystem functioning during the long-term restoration of reclaimed mining areas.

### 3.2. Influence Mechanisms of Land Use Types on Soil Microbial Diversity

Different land use types significantly altered soil bacterial diversity and community composition after six years of reclamation. Land use type significantly affected the Chao1 index, while soil depth and its interaction with land use had relatively weak effects (Table A2), indicating that vegetation type and management intensity were the primary drivers of bacterial diversity. PCoA and PERMANOVA further showed clear community differentiation among land use types, with land use explaining 26.4% of the variation in bacterial community composition (R^2^ = 0.264, *p* = 0.001). The Ligustrum plantation exhibited the most clustered bacterial communities and relatively high Simpson diversity, suggesting greater community stability, which may be associated with continuous carbon inputs from woody roots and litter and reduced soil disturbance [48,49,50,51]. In contrast, bacterial communities in the Vegetable field and Abandoned grassland were more dispersed and clearly separated from other treatments. Frequent tillage, fertilization, and irrigation in the Vegetable field likely increased fluctuations in resource availability and environmental selection pressures, promoting microbial heterogeneity despite its relatively low SOC and TN contents [52]. The Abandoned grassland also showed distinct bacterial communities, which may reflect its low C and N availability and early successional stage. Limited plant biomass, litter inputs, and rhizosphere carbon supply during early restoration can constrain microbial development and promote distinct community assembly [41]. In contrast, the Wheat field and Poplar plantation showed substantial overlap in bacterial community composition, possibly because both provided relatively stable resource environments and comparable plant-derived carbon inputs. Crop residue incorporation under long-term wheat–maize rotation may partly compensate for agricultural disturbance, whereas the relatively simple vegetation structure of the Poplar plantation may generate microbial resource conditions similar to those in cropland. Overall, microbial community assembly was jointly regulated by plant functional traits, carbon inputs, resource availability, and disturbance intensity, rather than by land use type alone [48,49].

Beyond shifts in community composition, this study further revealed the regulatory mechanisms of soil resources and heavy metals on microbial diversity. Correlation analysis showed that the Chao1 index was significantly associated with TN and As. Furthermore, SEM demonstrated that both soil nutrient availability and metal accumulation significantly affected bacterial richness (Figure 6). Specifically, soil nutrient levels showed a negative effect on Chao1 (β = −0.401, *p* = 0.035), whereas heavy metals exhibited a positive effect (β = 0.460, *p* = 0.015). These results indicate that microbial communities adopt differentiated response strategies to resource availability and environmental stress. The negative relationship between nutrient availability and bacterial richness suggests that increased soil resources do not necessarily promote microbial diversity. Instead, the effects of nutrient enrichment may be regulated by niche competition and resource filtering processes. Classical ecological theory suggests that higher resource availability generally increases microbial biomass and diversity. However, recent studies have shown that excessive nutrient inputs may reduce microbial diversity, particularly in ecosystems experiencing long-term nitrogen and phosphorus enrichment [53,54]. This reduction may occur because resource enrichment enhances the competitive ability of dominant microbial taxa and reduces differences in resource availability among microsites. As a result, microbial communities become more similar in composition, with oligotrophic taxa being excluded and copiotrophic taxa becoming increasingly dominant. This process, referred to as niche homogenization, reduces the number of distinct ecological niches available to microbial taxa and may ultimately decrease bacterial richness [54]. The negative effect of soil nutrients on Chao1 may reflect a shift in microbial community assembly from a resource-limited strategy toward a competition-driven selection strategy under higher nutrient availability. This finding highlights that nutrient enrichment can reshape microbial diversity not only through increased resource supply but also through changes in competitive interactions and ecological filtering processes.

SEM further showed that heavy metal accumulation had a positive effect on the Chao1 index, which differs from the conventional view that heavy metal toxicity generally reduces microbial diversity [55]. In this study, the detected metal levels were likely insufficient to induce substantial toxic effects on soil microorganisms. At concentrations far below toxicity thresholds, certain metals may serve as trace nutrients or stimulate microbial activity through hormesis effects, thereby enhancing metabolic processes and ecological niche differentiation [29,55,56]. As a result, bacterial richness may increase under moderate metal exposure. Moderate levels of metal availability may generate new environmental filtering gradients, increase habitat heterogeneity, and promote niche differentiation among metal–tolerant microbial taxa, thereby maintaining higher bacterial richness. However, when metal concentrations exceed ecological thresholds, toxic stress can reduce sensitive microbial groups and ultimately decrease microbial diversity [57,58]. Therefore, the effects of heavy metals on microbial communities are likely dose-dependent rather than uniformly negative. The positive relationship between heavy metals and Chao1 observed in this study may therefore reflect ecological filtering under low-level metal stress rather than contamination-induced toxicity. In addition, significant correlations were observed between soil nutrients and heavy metals. The SEM results showed that nutrients had a significant positive effect on heavy metals (β = 0.606, *p* < 0.001), indicating that nutrient accumulation induced by land use changes simultaneously promoted metal enrichment in soils. This coupled nutrient–metal variation may further influence microbial community assembly. Increased organic matter and nutrient availability can enhance soil metal adsorption capacity and provide more attachment sites for microorganisms. Meanwhile, metal-driven selection pressure may favor specific microbial groups with adaptive traits (e.g., increase metal resistance genus) [59,60], resulting in a complex resource–stress co-regulation framework. Furthermore, variance partitioning analysis showed that the marginal R^2^ values of endogenous variables ranged from 11% to 58%, whereas conditional R^2^ values reached 62–88%. This indicates that random effects at the site level explained a considerable proportion of the observed variation. Such effects may include microtopographic differences, residual coal gangue distribution, and local hydrological conditions. These findings highlight the inherent spatial heterogeneity of reclaimed mining soils and emphasize the importance of considering small-scale environmental factors in future studies investigating soil–microbial interactions.

Overall, this study revealed the coupled relationships among soil nutrient cycling, heavy metal accumulation, and microbial diversity under different land use types, providing further insights into the effects of land use change on belowground ecological processes. Previous studies have mainly focused on the impacts of land use conversion on individual ecosystem functions, such as soil carbon storage, nutrient availability, or microbial community structure. However, the interactions between soil resource availability and potential contamination risks have received relatively limited attention. Land use conversion does not simply alter soil nutrient levels; rather, it regulates microbial ecological strategies by creating distinct resource–stress combinations. The Ligustrum plantation primarily promoted the establishment of a stable microbial environment through long-term carbon inputs. In contrast, agricultural systems, including the Wheat field and Vegetable field, altered microbial community structure through nutrient inputs and soil disturbance. The Abandoned grassland remained constrained by limited resource availability during the early stage of ecological recovery. The SEM results further demonstrated that soil nutrients and heavy metals acted as key mediators linking land use changes with microbial diversity. Therefore, assessments of the ecological consequences of land-use change should consider the integrated effects of resource enrichment, contamination risks, and microbial adaptation processes, rather than relying on individual environmental factors alone. This integrated perspective is essential for developing effective management strategies for reclaimed mining ecosystems.

## 4. Materials and Methods

### 4.1. Experimental Site

The study area is located in an abandoned coal mining area between Qingshishan, No. 11 Coal Mine, and Xiangshan Coal Mine in Pingdingshan City, Henan Province, China (Figure 1). The region experiences a warm temperate continental monsoon climate, with a mean annual temperature of 15.0 °C and an average annual precipitation of approximately 755.7 mm. Precipitation is mainly concentrated from June to August. The landscape is characterized by low mountains and hills, with elevations ranging from 114 to 123 m. Long-term coal mining activities have resulted in extensive land degradation, including the formation of subsidence pits, coal gangue piles, and transportation-related pollution sites. Ecological restoration was implemented through coal gangue backfilling and topsoil covering, with an average soil cover thickness of approximately 20 cm. The regional soil is predominantly classified as Cinnamon soil, which represents the main background soil type in the study area. Since 2017, local authorities and residents have established vegetation restoration sites under different land-use practices. Soil sampling was conducted six years after reclamation, representing an early reclamation stage with established vegetation and developing soil properties. This timing also minimized potential bias caused by subsequent land-use transitions, particularly the conversion of non-agricultural reclaimed land to cropland.

### 4.2. Experimental Design and Soil Sample Collection

In May 2023, five land-use types were selected within the reclaimed mining area: (1) abandoned grassland, dominated by *Cirsium arvense var. integrifolium* Wimm. & Grab., *Humulus scandens* (Lour.) Merr., *Setaria viridis*, *Chenopodium album* L., and *Digitaria sanguinalis* (L.) Scop., with no artificial management and representing natural vegetation recovery; (2) cropland, under a winter wheat–summer maize rotation system, with conventional tillage to a depth of 15 cm and an average annual application of compound fertilizer at 450 kg ha^−1^; (3) poplar plantation (*Populus nigra* L.), established with a planting spacing of 2 m × 2 m, a stand age of 6 years, and no understory tillage; (4) privet plantation (*Ligustrum lucidum*), established with a planting spacing of 3 m × 3 m and a stand age of 6 years; and (5) vegetable field, characterized by highly intensive management, approximately 20 irrigation events per year, and combined application of organic and chemical fertilizers.

For each land use type, five independent plots (2 m × 2 m) were established. The plots were distributed across reclaimed platforms located between two abandoned mine sites and two coal gangue waste piles. The distance between adjacent plots was greater than 50 m to ensure spatial independence (Figure 1 and Table A1). Undisturbed soil cores and composite soil samples were collected from two soil layers (0–10 cm and 10–20 cm) at each plot. Undisturbed soil cores were collected using stainless-steel rings for the determination of soil bulk density and porosity. Composite soil samples were carefully cleaned to remove visible roots and gravel, air-dried, and passed through a 2 mm sieve for subsequent analyses. A total of 50 soil samples were obtained, including five land use types × five replicates × two soil depths.

### 4.3. Soil Physicochemical Properties and Heavy Metal Determination

Soil moisture content was determined using the oven-drying method at 105 °C. Soil bulk density and soil porosity, including total porosity (TPO), capillary porosity (CP), and non-capillary porosity (NCP), were measured using the core-ring method. Soil pH was determined using a pH meter (HI98130, Hanna Instruments, Woonsocket, RI, USA) after extraction with distilled water at a soil-to-water ratio of 1:2.5.

Soil total carbon (TC) and TN concentrations were analyzed using an elemental analyzer (Vario MACRO cube, Elementar, Langenselbold, Germany). TP was determined by sulfuric acid–perchloric acid digestion, followed by molybdenum–antimony colorimetric analysis. SOC was measured using the potassium dichromate external heating oxidation method. Based on the measured SOC, TN, and TP concentrations, the soil ecological stoichiometric ratios of C:N, C:P, and N:P were calculated.

Soil ammonium nitrogen (NH_4^+^_-N) and nitrate nitrogen (NO_3^−^_-N) were extracted with 2 mol L^−1^ KCl solution at a soil-to-solution ratio of 1:10 (m:v). The concentrations of NH_4^+^_-N and NO_3^−^_-N in the extracts were determined by spectrophotometric methods. Specifically, NH_4^+^_-N was quantified using the indophenol blue colorimetric method, whereas NO_3^−^_-N was measured using ultraviolet spectrophotometry (UV-1800PC, Shanghai, China). Soil available phosphorus (AP) was extracted using sodium bicarbonate solution (NaHCO_3_, pH 8.5), and phosphorus concentrations in the extracts were determined using the molybdenum–antimony colorimetric method. The total concentrations of heavy metals, including Cd, Pb, Cr, As, Mn, Cu, Zn, Ni, and Hg, were determined after digestion with HNO_3_, H_2_O_2_, and HF at a volume ratio of HNO_3_:H_2_O_2_:HF = 5:2:1 (*v v*^−1^). Briefly, 0.1 g of soil sample was pre-digested overnight with 5 mL of concentrated HNO_3_, followed by the addition of 2 mL of H_2_O_2_ and 1 mL of HF. The samples were digested at 150–170 °C for 4 h and subsequently heated at 160 °C for 30 min. The resulting solutions were diluted to 25 mL with 1% HNO_3_ before instrumental analysis. The digested solutions were analyzed using inductively coupled plasma mass spectrometry (ICP-MS, NexION^®^ 1000, PerkinElmer, Waltham, MA, USA). The analytical accuracy was evaluated using the certified reference material GBW07407. The recoveries of the target metals ranged from 90.96% to 110.67%, indicating satisfactory accuracy and reliability of the analytical procedure. Procedural blanks and replicate analyses were conducted simultaneously to ensure analytical precision and accuracy.

### 4.4. Bacterial Diversity Analysis

Fresh soil samples (0.5 g) were used for total DNA extraction using the E.Z.N.A.^®^ Soil DNA Kit (Omega Bio-tek, Norcross, GA, USA) according to the manufacturer’s instructions. The integrity and purity of extracted DNA were evaluated by 1% agarose gel electrophoresis, and DNA concentration and purity were quantified using a NanoDrop One spectrophotometer (Thermo Fisher Scientific, Wilmington, DE, USA). The bacterial 16S rRNA gene V3–V4 hypervariable region was amplified using genomic DNA as the template with the barcode-containing universal bacterial primers. PCR amplification was performed using Premix Taq (TaKaRa Bio Inc., Kusatsu, Shiga, Japan). Sequencing libraries were constructed following the standard protocol of the NEBNext^®^ Ultra™ DNA Library Prep Kit for Illumina^®^ (New England Biolabs, Ipswich, MA, USA). The qualified libraries were subsequently sequenced on an Illumina NovaSeq 6000 platform (Illumina, San Diego, CA, USA) using paired-end 250 bp sequencing. Raw image data generated from sequencing were processed by base calling and converted into raw sequence reads in FASTQ format. The sequences were clustered into operational taxonomic units (OTUs) based on 97% sequence similarity using the UPARSE algorithm. Bacterial alpha diversity indices, including Chao1 richness, Shannon diversity, and Simpson diversity, were calculated using R software (version 4.6.1).

### 4.5. Data Processing and Statistical Analysis

Two-way analysis of variance (two-way ANOVA) was performed to evaluate the effects of land-use type, soil depth, and their interactions on soil physicochemical properties, heavy metal concentrations, and bacterial diversity. One-way ANOVA was used to determine the significance of differences in soil properties, heavy metal contents, and microbial diversity among different land-use types within each soil layer. Data that met the assumption of homogeneity of variance were further analyzed using the least significant difference (LSD) test. For variables that violated the assumption of homogeneity of variance, differences among land use types were assessed using the non-parametric Kruskal–Wallis test. All statistical analyses were conducted using SPSS 25.0 (International Business Machines Corporation [IBM], Armonk, NY, USA). Bar plots illustrating soil nutrients, ecological stoichiometric ratios, heavy metal concentrations, and bacterial alpha diversity indices were generated using Origin 2025b (OriginLab Corporation, Northampton, MA, USA). Analyses of α and β diversity (principal coordinate analysis (PCoA) based on Bray–Curtis), principal component analysis (PCA), correlation heatmaps, and structural equation modeling (SEM) were performed in R software (version 4.6.1). Soil nutrient variables, physical properties, and heavy metal variables were separately standardized using Z-score transformation. Independent PCA analyses were conducted for each variable group, and sample ordination plots with 95% confidence ellipses, together with variable loading biplots, were generated. Correlation heatmaps were constructed using the ggcorrplot package (version 0.2.0).

To quantify the direct and indirect effects of land use type, soil depth, and soil properties on heavy metal distribution and bacterial diversity, a piecewise structural equation model (piecewise SEM) was developed. Prior to model construction, PCA was performed separately on soil basic properties (bulk density, pH, and soil moisture content), nutrient variables (soil organic carbon, total nitrogen, and total phosphorus), and heavy metal concentrations (Cd, Pb, As, and Hg) to reduce dimensionality and minimize multicollinearity. The first principal component (PC1) from each PCA was extracted as an integrated indicator representing the corresponding latent variable. Land use type was treated as a categorical exogenous variable, and the path model was established based on ecological hypotheses. All component models were fitted using linear mixed-effects models (LMMs), with sampling site (Site) included as a random intercept term (1 | Site) to account for potential spatial pseudoreplication effects.

## 5. Conclusions

This study demonstrated that land use types strongly shaped soil elemental cycling and bacterial diversity after reclamation. Agricultural and woody land uses showed distinct patterns of soil C, N, P, and heavy metal accumulation, reflecting differences in nutrient inputs, plant-derived carbon inputs, and soil disturbance. Structural equation modeling revealed that soil nutrient status positively promoted heavy metal accumulation (β = 0.606, *p* < 0.001), while nutrients negatively affected bacterial richness (β = −0.401, *p* = 0.035) and heavy metals exerted a positive effect (β = 0.460, *p* = 0.015), highlighting the coupled effects of resource availability and environmental filtering on microbial communities. The Wheat field and Vegetable field enhanced nutrient availability through intensive management, whereas the Ligustrum and Poplar plantations promoted organic matter accumulation through sustained plant inputs but may also facilitate heavy metal retention. These findings highlight the need to integrate nutrient management and heavy metal risk assessment into reclamation strategies. Long-term field monitoring with microbial functional analyses is needed to clarify how land use legacies regulate soil elemental cycling and microbial functions over time.

## Figures and Tables

**Table 1:** Differences in soil physical properties, water content, and pH among five land use types.

Land Use Types	Soil Depth (cm)	Moisture Content (%)	Bulk Density (g cm^−3^)	Total Porosity (%)	Capillary Porosity (%)	Non-Capillary Porosity (%)	pH
Abandoned grassland	1–10	6.679 ± 1.466 c	1.498 ± 0.092 a	43.892 ± 4.129 b	39.240 ± 3.381 a	4.652 ± 0.887 a	8.128 ± 0.017 a
	10–20	9.847 ± 1.795 B	1.512 ± 0.081 AB	40.670 ± 2.170 BC	36.582 ± 1.301 BC	4.088 ± 1.252 AB	8.154 ± 0.022 A
Wheat field	0–10	15.087 ± 2.016 a	1.418 ± 0.075 ab	44.796 ± 1.924 ab	40.362 ± 0.978 a	4.434 ± 1.364 a	8.130 ± 0.036 a
	10–20	16.670 ± 1.386 A	1.562 ± 0.028 A	38.454 ± 0.884 C	36.132 ± 0.876 C	2.322 ± 0.226 BC	8.144 ± 0.032 A
Poplar plantation	0–10	9.790 ± 0.979 bc	1.381 ± 0.051 ab	46.344 ± 0.907 ab	41.698 ± 0.488 a	4.646 ± 0.960 a	8.062 ± 0.061 a
	10–20	10.288 ± 1.103 B	1.362 ± 0.053 BC	46.986 ± 0.819 A	42.356 ± 1.143 A	4.630 ± 1.164 AB	8.138 ± 0.053 A
Vegetable field	0–10	8.709 ± 1.421 bc	1.267 ± 0.017 b	50.602 ± 0.605 a	42.718 ± 2.316 a	7.884 ± 1.848 a	8.064 ± 0.050 a
	10–20	12.918 ± 2.132 AB	1.359 ± 0.016 C	44.608 ± 0.342 AB	38.046 ± 0.744 BC	6.562 ± 0.627 A	8.084 ± 0.043 A
Ligustrum plantation	0–10	12.690 ± 0.901 ab	1.255 ± 0.039 b	48.602 ± 0.616 ab	42.760 ± 1.093 a	5.842 ± 0.743 a	8.172 ± 0.012 a
	10–20	14.116 ± 0.599 AB	1.384 ± 0.052 BC	44.746 ± 1.746 A	39.484 ± 1.089 AB	5.262 ± 1.743 AB	8.158 ± 0.019 A

Note: Mean ± standard error, *n* = 5. Different lowercase letters indicate significant differences among land use types in the 0–10 cm soil layer, whereas different uppercase letters indicate significant differences among land use types in the 10–20 cm soil layer (*p* < 0.05).

## Data Availability

The original contributions presented in this study are included in the article. Further inquiries can be directed to the corresponding author.

## Abbreviations

The following abbreviations are used in this manuscript:

AG	Abandoned grassland
WF	Wheat field
PP	Poplar plantation
VF	Vegetable field
LP	Ligustrum plantation

## Appendix A

**Table A1:** Sampling points and corresponding land use types for Figure 1.

Sampling Point	Land Use Types
1	Abandoned grassland
2	Wheat field
3	Poplar stand
4	Abandoned grassland
5	Poplar stand
6	Wheat field
7	Ligustrum stand
8	Ligustrum stand
9	Wheat field
10	Ligustrum stand
11	Wheat field
12	Abandoned grassland
13	Ligustrum stand
14	Wheat field
15	Poplar stand
16	Poplar stand
17	Poplar stand
18	Vegetable field
19	Vegetable field
20	Vegetable field
21	Abandoned grassland
22	Vegetable field
23	Ligustrum stand
24	Vegetable field
25	Abandoned grassland

**Table A2:** Effects of land use types, soil depth and their interaction on soil physical and chemical properties.

Parameters	Land Use Types		Soil Depth		Land Use Types × Soil Depth	
	*F*	*p*	*F*	*p*	*F*	*p*
Moisture content (%)	8.397	**<0.001**	5.570	**0.023**	0.520	0.721
Bulk density (g cm^−3^)	5.381	**0.001**	4.101	**0.050**	0.810	0.526
Total porosity (%)	4.920	**0.003**	11.136	**0.002**	1.238	0.310
Capillary porosity (%)	2.619	**0.049**	8.141	**0.007**	0.898	0.474
Non-capillary porosity (%)	3.012	**0.029**	1.514	0.226	0.237	0.916
pH	1.793	0.149	1.024	0.318	0.367	0.831
TC	0.257	**0.002**	0.311	0.580	0.093	0.984
TN	7.564	**<0.001**	7.990	**0.007**	1.064	0.387
TP	10.139	**<0.001**	0.569	0.455	0.235	0.917
SOC	2.215	0.085	0.818	0.371	0.255	0.905
C:N	0.466	0.760	0.544	0.465	0.014	1.000
C:P	0.044	0.996	0.421	0.520	0.244	0.911
N:P	1.092	0.373	7.716	**0.008**	1.105	0.368
NH^4+^-N	3.583	**0.014**	0.139	0.712	0.902	0.472
NO^3−^-N	5.148	**0.002**	0.305	0.584	0.085	0.987
aP	1.299	0.287	0.527	0.472	0.569	0.686
Cd	3.149	**0.024**	0.312	0.580	0.280	0.889
Pb	6.754	**<0.001**	1.238	0.272	0.232	0.919
Hg	0.228	0.921	0.014	0.908	0.763	0.556
Cr	3.554	**0.014**	2.654	0.111	0.641	0.636
As	3.351	**0.019**	0.979	0.328	0.349	0.843
Mn	2.477	0.059	0.099	0.755	0.056	0.994
Cu	0.785	0.542	1.228	0.274	0.315	0.867
Zn	1.271	0.297	0.003	0.959	0.258	0.903
Ni	3.263	**0.021**	0.986	0.327	0.439	0.779
Shannon	1.936	0.123	0.143	0.708	0.754	0.562
Simpson	0.992	0.423	0.813	0.373	0.926	0.458
Chao1	3.473	**0.016**	0.497	0.485	1.039	0.399

Note: Two-way ANOVA was used to evaluate differences in *p*-values (bold indicates significant differences between treatment levels at *p* < 0.05, *p* < 0.01, or *p* < 0.001).

**Table A3:** Piecewise structural equation model path coefficients (standardized).

Response	Predictor	Estimate	Std. Error	DF	Crit. Value	*p*-Value	Std. Estimate	Sig.
Basic_PC1	Types (overall)	–	–	4.000	2.7585	0.0550	–	
	Types = AG	−1.0064	0.3775	20.575	−2.6662	0.0146	–	*
	Types = PP	−0.0833	0.3775	20.575	−0.2207	0.8275	–	
	Types = WF	0.2101	0.3775	20.575	0.5566	0.5838	–	
	Types = VF	0.3335	0.3775	20.575	0.8836	0.3871	–	
	Types = LP	0.5907	0.3524	22.246	1.6765	0.1076	–	
	Depth (overall)	–	–	1.000	0.0187	0.8925	–	
	Depth = Sub	0	0.179	27.165	−0.0002	0.9999	–	
	Depth = Top	0.0179	0.179	27.165	0.0999	0.9212	–	
Nutrient_PC1	Basic_PC1	0.0515	0.127	41.829	0.1508	0.6997	–	
	Types (overall)	–	–	4.000	3.4147	0.0263	–	*
	Types = AG	−0.9156	0.3732	23.203	−2.4532	0.0221	–	*
	Types = VF	−0.4952	0.3513	20.404	−1.4097	0.1737	–	
	Types = LP	0.3252	0.333	22.554	0.9767	0.3391	–	
	Types = PP	0.4736	0.3487	20.064	1.3582	0.1895	–	
	Types = WF	0.6028	0.3496	20.185	1.7242	0.1000	–	
	Depth (overall)	–	–	1.000	13.1538	0.0015	–	**
	Depth = Sub	−0.1887	0.1621	24.803	−1.1644	0.2554	–	
	Depth = Top	0.185	0.1621	24.808	1.1414	0.2646	–	
Metal_PC1	Nutrient_PC1	0.6061	0.1363	27.277	18.5597	0.0002	–	***
	Basic_PC1	−0.1677	0.1216	30.913	1.7639	0.1939	–	
	Depth (overall)	–	–	1.000	2.5963	0.1186	–	
	Depth = Top	−0.133	0.1325	35.294	−1.0040	0.3222	–	
	Depth = Sub	0.1267	0.1325	35.237	0.9560	0.3456	–	
	Types (overall)	–	–	4.000	2.5234	0.0737	–	
	Types = AG	−0.3864	0.2902	20.195	−1.3313	0.1979	–	
	Types = VF	−0.2677	0.2515	19.150	−1.0646	0.3003	–	
	Types = PP	−0.183	0.2462	18.668	−0.7431	0.4667	–	
	Types = WF	0.1666	0.2514	18.773	0.6626	0.5156	–	
	Types = LP	0.6547	0.2435	21.701	2.6884	0.0135	–	*
Chao1	Nutrient_PC1	−0.4009	0.1785	45.919	4.7306	0.0348	−0.4009	*
	Metal_PC1	0.4596	0.1761	44.939	6.3822	0.0151	0.4596	*

Notes: (1) Crit. Value represents F-statistic for overall factor effects (Types, Depth) and t-statistic for individual contrasts and continuous predictors. (2) Significance codes: * *p* < 0.05, ** *p* < 0.01, *** *p* < 0.001. (3) Std. Estimate is available only for continuous predictor–response pairs. (4) The model’s Fisher’s C statistic indicated good overall fit (C = 1.124, df = 4, *p* = 0.89). For Depth, “Top” refers to 0–10 cm, “Sub” to 10–20 cm. Crit. Value is F-value for overall factor effects and t-value for individual contrasts. Basic_PC1, Nutrient_PC1 and Metal_PC1 are the first principal components of soil basic properties, soil nutrient contents and heavys, respectively. Chao1 represents soil microbial diversity.

**Table A4:** Marginal and conditional R^2^ for each component model in the piecewise SEM.

Response	Marginal R^2^	Conditional R^2^
Basic_PC1	0.276	0.816
Nutrient_PC1	0.386	0.883
Metal_PC1	0.585	0.720
Chao1	0.107	0.616

Note: Marginal R^2^ represents the variance explained by fixed effects (land use type, depth, and other continuous predictors), while conditional R^2^ includes both fixed and random effects (Site). All variables were Z-score standardized prior to analysis.

**Table A5:** Variance explained by the first principal component (PC1) derived from soil physical, nutrient, and heavy metal variables.

Variable Group	PC1 Standard Deviation	Proportion of Variance	Cumulative Proportion
Basic properties (BD, pH, MC)	1.0838	0.3916	0.3916
Nutrients (SOC, TN, TP)	1.5856	0.8381	0.8381
Heavy metals (Cd, Pb, As, Hg)	1.3415	0.4499	0.4499

**Table A6:** Variance components of random effects in the linear mixed-effects models.

Response	Random Effect	Variance	Std. Dev.
Basic_PC1	Site (Intercept)	0.609	0.780
	Residual	0.208	0.456
Nutrient_PC1	Site (Intercept)	0.543	0.737
	Residual	0.128	0.358
Metal_PC1	Site (Intercept)	0.137	0.370
	Residual	0.285	0.533
Chao1	Site (Intercept)	0.500	0.707
	Residual	0.378	0.615

Note: Random intercept models included Site as a random effect (1|Site) to account for spatial pseudoreplication. Basic_PC1, Nutrient_PC1 and Metal_PC1 are the first principal components of soil basic properties, soil nutrient contents and heavys, respectively. Chao1 represents soil microbial diversity.

**Table A7:** Risk screening values for soil heavy metal contamination in agricultural land specified in the Chinese Soil Environmental Quality–Risk Control Standard for Soil Contamination of Agricultural Land (Trial) (GB 15618–2018) [47].

Soil Heavy Metal	Risk Screening Values			
	pH ≤ 5.5	5.5 < pH ≤ 6.5	6.5 < pH ≤ 7.5	pH > 7.5
Cd	0.3	0.3	0.3	0.6
Hg	1.3	1.8	2.4	3.4
As	40	40	30	25
Pb	70	90	120	170
Cr	150	150	200	250
Cu	50	50	100	100
Ni	60	70	100	190
Zn	200	200	250	300
